# A Cough Symptom

**Published:** 1889-01

**Authors:** 


A COUGH SYMPTOM:—Cough, with hæmoptysis and feeble, weak heart action; deep, violent in evening and night, without waking; renewed by change to cold weather and by cold winds. Expectoration pale, sweetish, unpleasant tasting, at times difficult, renewed same as cough—Lycopus-virg.

